# *Cucurbita ficifolia* Fruit Extract Induces Tp53/Caspase-Mediated Apoptosis in MCF-7 Breast Cancer Cells

**DOI:** 10.1155/2020/3712536

**Published:** 2020-06-25

**Authors:** Ghedeir M. Alshammari, Aristatile Balakrishnan, Ali A. Alshatwi, Abdulrahman Al-Khalifa

**Affiliations:** Adipocytes and Metabolic Disorders Lab, Department of Food Science and Nutrition, College of Food and Agricultural Science, King Saud University, P.O. Box 2460, Riyadh 11451, Saudi Arabia

## Abstract

The second most biggest cancer worldwide is breast cancer. There is an increasing need for safer, effective, and affordable drug candidates from natural sources to treat breast cancer. In the present investigation, the anticancer effect of *Cucurbita ficifolia* Bouché (*C*. *ficifolia*) fruit extract was tested on the human breast cancer cells such as MCF-7. The cells were exposed with different doses of *C*. *ficifolia*, for the assessment of IC_50_ concentrations on the MCF-7 cell lines for 24 hs. The effect of *C*. *ficifolia* fruit extract on morphological and apoptotic changes were evaluated by specific fluorescence staining techniques and real-time PCR in a time-dependent manner for 24 hs and 48 hs. The IC_50_ value for *C*. *ficifolia* fruit extract was found to be 90 *μ*g/mL. Morphological alteration and apoptotic distinctiveness aspect like chromatin condensation and nuclear fragmentation were noticed in *C. ficifolia* extract exposed breast cancer cells. Further, we observed that *C*. *ficifolia* extract-induced programmed cell death in the MCF-7 cells were mediated with the elevated expression of the tumor suppressor gene such as p53 and apoptotic markers such as caspase-8, caspase-9, caspase-3, fatty acid synthase (FAS), Fas-associated protein with death domain (FADD), Bcl-2 homologous antagonist/killer (BAK), and Bcl-2-associated X protein (BAX). These observations established that *C*. *ficifolia* significantly concealed the cell division and provoked p53/caspase-mediated programmed cell death. Further, we noticed that this cell death in MCF-7 cells is concentration and time dependent. As evaluated through the comet assay, *C*. *ficifolia* induced DNA damage; further upon increasing the duration of the treatment, the DNA damage was higher than before. Thus, our study concludes that *C*. *ficifolia* could serve as an effective anticancer agent through vital gene modulation.

## 1. Introduction

The most widespread cancer around the world in women is breast cancer, with nearly 2 million fresh patients diagnosed in 2018. This data shows that ~12% of all new cancer patients and 25% of all cancers in women are breast cancer. On the one hand, the incidence of breast cancer is increasing every year; on the other hand, mechanisms leading to the progression of breast cancer are still in mercy. The molecular mechanisms and genetic alterations in this disease have not been completely established [1]. The occurrence of breast cancer is increasing in the developing world due to the increase in urbanization, unhealthy dietary habits, and adoption of Western lifestyles [2].

Numerous studies show that evasion of programmed cell death (PCD) is one of the properties acquired by cancer cells. On the other hand, research reports show that PCD is a widespread mechanism through which most of the cancer chemotherapeutic agents exert their anticancer effect [3]. Studies show that gene-regulating apoptosis can mediate chemosensitivity [4, 5]. Various research data report that p53 level could be one of the significant determinants for tumor chemosensitivity [6, 7]. It is known that ~50% of all human tumors have mutated tumor suppressor gene p53 (TSG-p53) [8]. Further, it is observed that loss of regular p53 function is implicated in hereditary as well as sporadic breast cancer. Because of the TSG mutation, breast cancer shows different response to conventional cancer drugs [9]. On the other hand, these cancer cells also develop drug resistance to normal drug therapy [10]. The low efficiency of present chemotherapeutic drugs has warranted new research into different foundations to progress existing therapy regime or to serve as a revenue of chemoprevention.

PCD is described by the commencement of precise cysteine proteases branded as caspases, chromatin compression, DNA disintegration, and cell contraction. Two apoptotic passageways congregate on caspase-3, the extrinsic pathway concerning caspase-8 activation, and the intrinsic pathway involving mitochondrial discharge of cytochrome c and commencement of caspase-9 activation [11]. Apoptosis is a key factor for the evaluation of potential agents for cancer treatment and prevention [12].

Almost all cancer cells show drug resistance and also lowered efficacy to the currently used chemotherapeutic substances; this urged all cancer researchers to search novel drugs from natural foundations. Even though the existing chemotherapeutic agents are talented to hinder or slaughter tumor cells, the problem of toxicity and adverse effects restrict the clinical usage of these chemicals as a drug candidate. A natural substance, which could slaughter the cancer cells and has nil or less toxicity on healthy cells, is considered a good candidate for cancer therapeutic strategies [13].

*Cucurbita ficifolia* Bouché (*C*. *ficifolia*), from the Cucurbitaceae family, is grown in the whole world as a food crop as well as a traditional medicine. Further, this plant has been used in China, Argentina, India, Brazil, and Iran as traditional medicine [14]. This plant has been acknowledged as a functional food because of its numerous medicinal application [15–17]. Further, anti-inflammatory cardiovascular and hepatoprotective substances have been isolated from this plant, and cytotoxicity was also established [18]. In addition, the mature fruit macerated in water has been used for the treatment of diabetes [19]. The antiobese effect has been reported in literature [20]. Recently, we have established its antilipogenic effect through mesenchymal stem cell transition [21].

As part of the larger project, the present study focused on the investigation of cytotoxicity of the chloroform extract of *C*. *ficifolia* in breast cancer MCF-7 cell lines. Besides studying the cytotoxicity in MCF-7 cells, the present study specifically analyzed the effect of the *C*. *ficifoli*a chloroform extract on Tp53/caspase-mediated apoptosis to find out the probable mechanism of cell death in MCF-7 cell lines. Further, we also assayed the effect of the *C*. *ficifolia* extract on normal rat hepatocytes.

## 2. Materials and Methods

### 2.1. Plant Material and Extraction

Cucurbita ficifolia Bouché (*C*. *ficifolia*) fruit extract was selected on the basis of ethnopharmacology. *C*. *ficifolia* fruit was collected from reputable farms in the month of April 2015, Riyadh, Saudi Arabia. The plant material was authenticated by Dr. Jacob Thomas Pandalayil, Herbarium Division, College of Science, King Saud University, Riyadh 11451 (no. KSU-7965), Saudi Arabia. The whole fruit excluding the outer shell was dried in a shaded place and ground and soaked in chloroform for extraction. The solvent amount was taken 10 times more than the amount of ground plant substance. Extraction was carried out three times for an overall time period of 24 hs. Under reduced pressure, the extract was allowed to evaporate to dryness at 30°C. For the experimental purpose, 100 mg of the extract was dissolved in 10 mL Dulbecco's Modified Eagle's Medium (DMEM) (10% FCS) to get a stock solution (10 mg/mL) and was additionally thinned with a medium to various concentrations ranging between 0 and 150 *μ*g/mL for the study. In our previous study, we have identified the active components present in the *C*. *ficifolia* fruit: D-glucopyranosylamine (67.8%), n-hexadecanoic acid (17.7%), and 1,4-cyclooctadiene (15.8%) as major components using GC-MS NIST library analysis [21].

### 2.2. Maintenance of MCF-7 Cells

The mycoplasma-free MCF-7 breast cancer cell line was used for the current investigation. The MCF-7 cell was cultured and allowed to grow in 90% DMEM + phenol red, complemented with 10% fetal bovine serum (FBS) and streptomycin/penicillin (0.1 mg/100 units, mL) in a humidified ambiance of 95% air and 5% CO_2_ at 37°C. Every single one of the experimental investigations was performed in cells grown to ~70% to 80% confluence. All the experiments were performed between the 3^rd^ and 5^th^ passages of MCF-7 cells. After the experimental periods, the cells were collected following trypsinization. The Trypan blue exclusion test was used to assay cell viability. The control cell viability was found to be superior than 95%.

### 2.3. CellTiter-Blue® Viability Assays

In MCF-7 cells, CellTiter-Blue® Viability Assay (Promega) was carried out to evaluate the toxicity of various doses of the *C*. *ficifolia* extract. The manufacturer's directions were exactly followed to carry out the experiment. In detail, MCF-7 cells (2 × 10^4^ cells/well) were grown in 96-well cell culture plates and exposed with 0-150 *μ*g/mL of extract for 24 hs. After the experimental period, 40 *μ*L of cell titer blue viability solution was straightway added to the experimental cells, and incubation was continued further for 6 hs at 37°C. Using a Bio-Rad microplate fluorescence reader, the fluorescence intensity was recorded at 560 nm excitation and 590 nm emission filter. IC_50_ was derived from the above said reading. Quadruplet samples were run for every experimental doses of the *C*. *ficifolia* extract in three autonomous experiments. The results were expressed as mean ± SD.

(1) $$\begin{array}{c} \text{Viability }\left(\%\right)={\left[A\right]}_{\text{test}}/{\left[A\right]}_{\text{control}}\times 100, \end{array}$$

where [*A*]_test_ is the absorbance of the test sample and [*A*]_control_ is the absorbance of a control sample.

### 2.4. Studies on Normal Hepatocytes

Hepatocytes were isolated from a healthy rat and maintained as explained by Subastri et al. [22]. The cells were exposed to *C*. *ficifolia* extract (0-150 *μ*g/mL) for 24 hs. After the experimental period, cell viability was assayed as mentioned above.

### 2.5. TUNEL Assay

To study the concentration- and time-dependent effect of the *C*. *ficifolia* extract on apoptosis, the DeadEnd® TUNEL Assay kit (Promega) was employed. The manufacturer's directions were stringently followed. In detail, cells (MCF-7 cells (1.5 × 10^6^ cells/well)) were subcultured a 6-well plate and allowed to grow for semiconfluence. Cells were exposed to a fresh medium containing the *C*. *ficifolia* extract at a concentration of 90 *μ*g/mL for 24 hrs and 48 hrs. Subsequent to the experimental duration, the culture medium containing the *C*. *ficifolia* extract was removed, and experimental cells were washed twice with ice-cold PBS. Cells were fixed with 4% methanol-free formaldehyde solution. After fixing, cells were again washed with normal PBS (twice) and stained according to the DeadEnd fluorometric TUNEL scheme procedure. Control and experimental cells stained with TUNEL assay solutions were observed under a Carl-Zeiss (Axiovert) epifluorescence microscope using a triple bandpass filter. Each condition minimum of ten random fields consisting around 1000 cells was counted and percent apoptotic positive cells were calculated. For calculation purpose, a person who is blind of the experimental condition was employed.

### 2.6. Acridine Orange/Ethidium Bromide (AO/EB) Staining

To distinguish apoptotic and necrotic cells, AO/EB staining procedure was adopted as explained by Subastri et al. [23]. In brief, MCF-7 cells were grown in 24-well cell culture plates (10^5^ cells/well). After noticing semiconfluence, cells were exposed to the *C*. *ficifolia* extract (90 *μ*g/mL) for 24 hrs and 48 hrs. After the experimental period, the cells were washed with PBS and stained with AO/EB dye mix (100 *μ*g/mL of AO and 100 *μ*g/mL of EB in PBS) for 5 min. Stained cells were washed with PBS to remove excess staining solution. The necrotic, apoptotic, and healthy cells were distinguished under the fluorescent microscope at 400x magnification.

### 2.7. Quantitative PCR Analysis through Real Time

Differences in key apoptotic genes were analyzed in the control and experimental cells. For the above said purpose, we have used the reverse transcription-PCR (RT-PCR) from Applied Biosystems 7500 Fast. Further, a real-time SYBR Green/ROX gene expression assay kit from QIAGEN was employed. Fastlane® Cell cDNA kit from QIAGEN was used to make the cDNA from different experimental cells. The mRNA levels of caspase-8, caspsase-3, caspase-9, protein-53 (p53), FAS, Fas-associated death domain (FADD), Bcl-2-associated X protein (BAX), and Bcl-2 homologous antagonist killer (BAK) as well as the reference gene glyceraldehyde-3 phosphate dehydrogenase (GAPDH) were assayed in different experimental conditions. We used gene-specific SYBR Green-based QuantiTect® Primer assays from QIAGEN. For quantitative real-time RT-PCR, the manufacturer's instruction was followed to make a reaction volume of 25 *μ*L. This mixture contains a master mix of 12.5 *μ*L, primer volume (2.5 *μ*L) (10x), and 100 *μ*g of template cDNA (10 *μ*L) added to every condition. Following a tiny centrifugation, the PCR plate was proceeded to 35 cycles of PCR reaction. The reaction cycle is as follows: the PCR activation at 95°C for 5 minutes, denaturation at 95°C for 5 seconds, and annealing/extension at 60°C for 10 seconds. Control and experimental conditions were run in triplicates on an ABI 7500 Fast Real-time PCR system. After the PCR cycle, the quantitative RT-PCR data was analysed. The comparative threshold (Ct) technique and the fold inductions of the test were evaluated against the healthy untreated cells. For each and every condition, the GAPDH expression was quantified and served as an internal reference gene. The expression profile of different apoptotic genes was normalised against the GAPDH gene. Further, the expression pattern of different apoptotic genes in the healthy control and *C*. *ficifolia* extract-exposed cells for 24 hrs and 48 hrs was calculated through the Ct value. The data were mentioned as the ratio of the GAPDH gene to objective the gene by using the subsequent formula: *∆*Ct = Ct (apoptotic genes) − Ct (GAPDH). To estimate the comparative expression patterns, the subsequent formula was employed: *∆∆Ct* = *∆Ct* (*treated*) − *∆Ct* (*control*). In crisp, the expression patterns were mentioned as *n*-level differences comparative to that of the calibrator. The calculated data was used to plot the expression of apoptotic genes using the expression of 2^-*∆∆*Ct^ [24].

### 2.8. DNA Damage by Comet Analysis

To estimate DNA damage in individual cells, the comet procedure was adopted as mentioned in Suyavaran et al., 2015.

### 2.9. Statistical Analysis

One-way analysis of variance (ANOVA) was used to calculate statistical differences. The different experimental samples were compared by Duncan's multiple range test (DMRT) using SPSS Software Package, version 11.0. Data were mentioned as mean ± S.D. (*n* = 6 in each sample). A value of *P* ≤ 0.05 was considered to be statistically significant.

## 3. Results

### 3.1. Effect of C. ficifolia Extract on Cell Cytotoxicity in MCF-7 Cells

**Figure 1:**
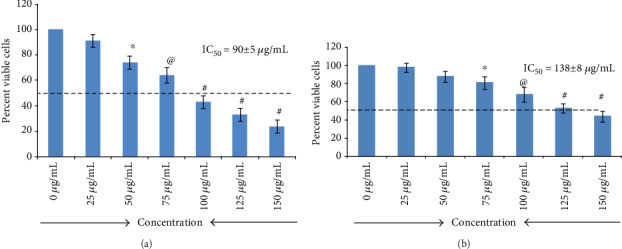
MCF-7 (a) and normal hepatocyte (b) cell viability was determined by the CellTiter assay. MCF-7 cells were treated with various concentrations (0-150 *μ*g/mL) of *C*. *ficifolia* chloroform extract and results are expressed as percentage of viability, normalized with untreated control (mean ± SD). ^∗^*P* ≤ 0.05, ^@^*P* ≤ 0.01, and ^#^*P* ≤ 0.001, control vs. different treatments. The experiment was repeated thrice with triplicate.

The CellTiter-Blue® Viability Assay was employed to formulate a preliminary estimation of the cytotoxicity effect of the *C*. *ficifolia* chloroform extract on MCF-7 cells. Further, to evaluate the individual IC_50_ at different doses of the *C*. *ficifolia* extract (0-150 *μ*g/mL), cells were exposed for 24 hrs and 48 hrs. A concentration-dependent decrease in cell viablity was noticed after 24 hr exposure to the *C*. *ficifolia* extract, and the IC_50_ of the *C*. *ficifolia* extract for MCF-7 cells was found to be 90 ± 5 *μ*g/mL (Figure 1(a)). Normal hepatocytes were exposed to different concentrations of the *C*. *ficifolia* extract. We found that IC_50_ of the *C*. *ficifolia* extract for hepatocytes is 130 ± 8 *μ*g/mL (Figure 1(b)). *P* < 0.05 significance was observed at 75 *μ*g/mL; the concentration of *C*. *ficifolia* at 24 hrs was 50 *μ*g/mL at 48 hrs, indicating that when the duration of exposure increased, the concentration of the extract required to inhibit cell growth is (*P* < 0.01 and *P* < 0.001) decreased. This result indicate that C. *ficifolia* extract more sensitive for MCF-7 cells.

### 3.2. Effect of C. ficifolia Extract on Apoptosis-Mediated Cell Death through TUNEL Assay

**Figure 2:**
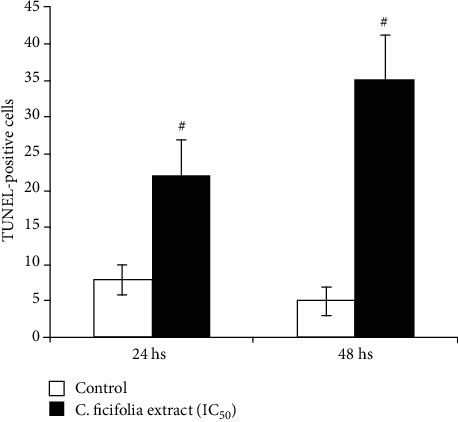
Percentage of TUNEL-positive cell indication of apoptosis after 24 hs and 48 hs of exposure of MCF-7 cells with or without *C*. *ficifolia* chloroform extract (90 *μ*g/mL). ^#^*P* ≤ 0.001, control vs. treatment. The experiment was repeated thrice with triplicate.

**Figure 3:**
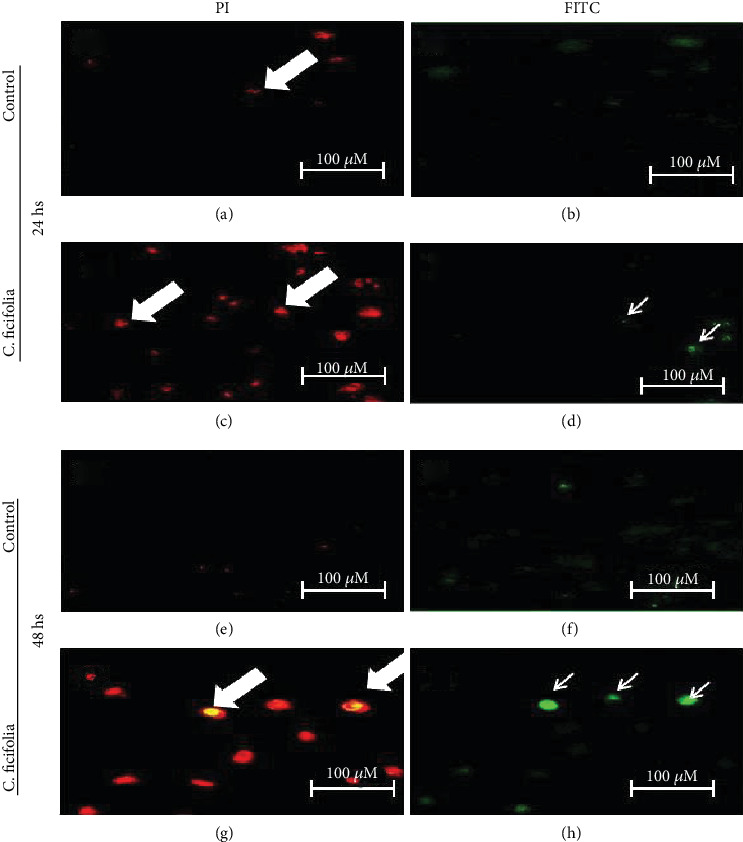
PI staining and TUNEL assay (microscopic) after 24 hs (a–d) and 48 hs (e–h) incubation of MCF-7 treated with 90 *μ*g/mL C. *ficifolia* chloroform extract with the control. Red fluorescence is due to propodium iodide (PI) (thick arrow) staining observed under green filter. TUNEL-positive cells (thin arrow) are with red fluorescence and blue indicates the nucleus. Observations were done at 200x for PI and 400x for TUNEL magnification.

The apoptotic effect of the *C*. *ficifolia* extract on MCF-7 cells was shown in Figures 2 and 3. We have used the TUNEL assay method to assess the *C*. *ficifolia* extract-induced apoptotic cell death. In the exposure of MCF-7 cells to the *C*. *ficifolia* extract, a time-dependent elevation in the induction of apoptosis was noticed. The IC_50_ dose (90 *μ*g/mL) of *C*. *ficifolia* extract-exposed cells shows 27 ± 4% (*P* < 0.001) TUNEL positive at 24 hrs, when compared with untreated healthy control cells. When the duration of the *C*. *ficifolia* extract exposure was increased, we have observed 42 ± 9% (*P* < 0.001) TUNEL-positive cells at 48 hrs when compared with normal cells.

### 3.3. Acridine Orange/Ethidium Bromide Staining

**Figure 4:**
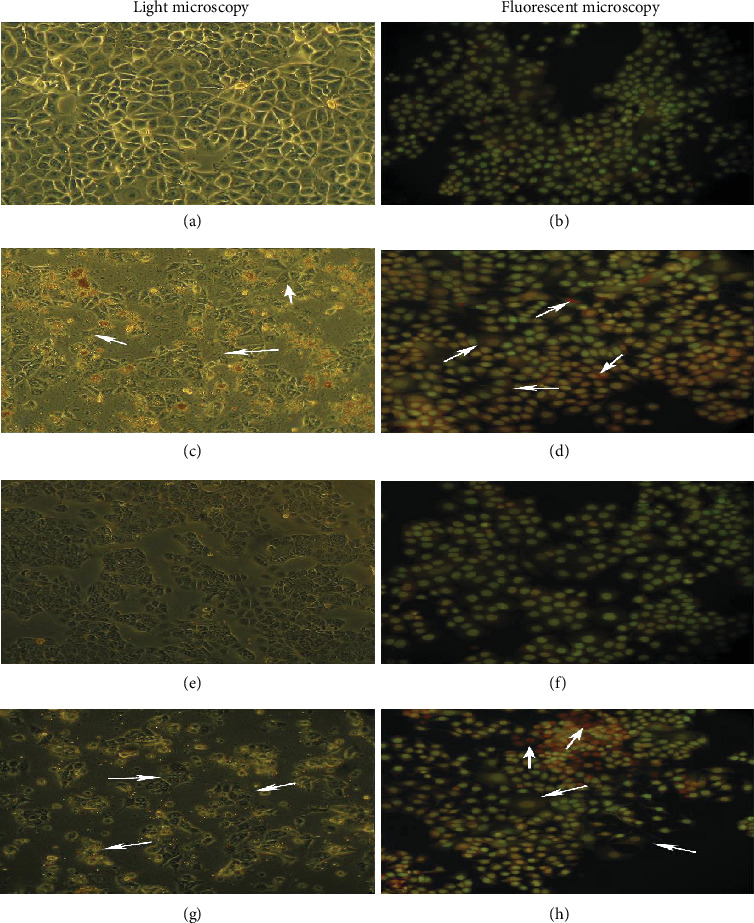
Phase contrast and fluorescent microscopy of MCF-7 cells treated with *C*. *ficifolia* chloroform extract after 24 hs (a–d) and 48 hs (e–h). Cell morphology is intact in untreated controls (a, c). Distortion of cell morphology and increased number of dead cells are evident from phase contrast microscopy (arrows in b and d). Early and late apoptotic cells are visible through AO/EB fluorescent staining (e–h). Nuclear fragmentation and ballooning can be observed, and the fragmentation of the nuclei and micronucleus is visible (arrows in f and h). Observation at 400x magnification.

AO/EB staining was employed to investigate the induction of apoptotic nuclear damage in the cytotoxicity of the *C*. *ficifolia* extract on MCF-7. Microscopic observations of cells stained with AO/EB indicated that untreated cells (negative control) displayed normal nuclear morphology with a bright green fluorescence. Whereas the cells treated with the *C*. *ficifolia* extract for 24 hrs and 48 hrs showed early apoptotic cells with greenish yellow nuclei, late apoptotic cells indicated condensed orange-red nuclei, while dead cells depicted red nuclei. Nuclear fragmentation, presence of apoptotic bodies, chromatin condensation, and membrane blabbing of apoptotic cells were also obvious upon assessment of AO/EB-stained cells under a fluorescent microscope (Figure 4).

### 3.4. Effect of C. ficifolia Extract on Quantification of mRNA Levels of Apoptotic-Related Genes

**Figure 5:**
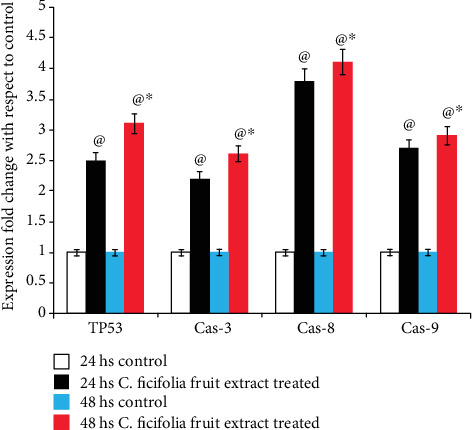
Comparison of the change in the expressions of p53, caspase-3, caspase-8, and caspase-9 genes expressed as respective gene/GAPDH (ratio) in MCF-7 cells after 24 hs and 48 hs of exposure with *C*. *ficifolia* chloroform extract (90 *μ*g/mL). ^@^*P* ≤ 0.001, respective control vs. treated samples. ^∗^*P* ≤ 0.05, 24 hs treatment of samples vs. 48 hs treatment. The experiment was repeated thrice with triplicate.

**Figure 6:**
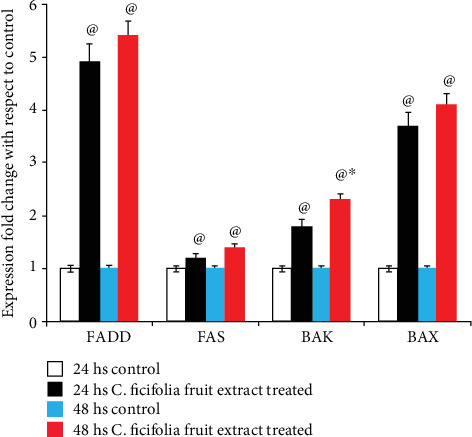
Comparison of the change in the expression of FADD, FAS, BAK, and BAX genes expressed as the fold change (ratio of target: reference gene) in MCF-7 cells after 24 hs and 48 hs of exposure with chloroform *C*. *ficifolia* extract (90 *μ*g/mL). ^@^*P* ≤ 0.001, respective control vs. treated samples. ^∗^*P* ≤ 0.05, 24 hs treatment of samples vs. 48 hs treatment. The experiment was repeated thrice with triplicate.

To investigate the molecular mechanism of *C*. *ficifolia* extract-induced apoptosis in MCF-7 cells, the expression levels of various apoptosis-related genes were examined by real-time PCR. Figures 5 and 6 summarize the *C*. *ficifolia* extract-induced apoptotic gene expression levels in the fold change of p53, caspase-3, caspase-8, and caspase-9 and FADD, FAS, BAK, and BAX in MCF-7 cells as compared to the control. The upregulated expression of p53 (*P* < 0.001, vs. the respective control; 24 hrs vs. 48 hrs, *P* < 0.05), caspase-3 (*P* < 0.001 vs. the respective control; 24 hrs vs. 48 hrs, *P* < 0.05), caspase-8 (*P* < 0.001 vs. the respective control; 24 hrs vs. 48 hrs, *P* < 0.05), and caspase-9 (*P* < 0.001 vs. the respective control; 24 hrs vs. 48 hrs, *P* < 0.05), genes in MCF-7 cells treated with 90 *μ*g/mL of the *C*. *ficifolia* extract for 24 and 48 hs (Figure 5). Similarly, the expression levels of FADD (*P* < 0.001 vs. the respective control; 24 hrs vs. 48 hrs, *P* < 0.05), FAS (*P* < 0.001 vs. the respective control; 24 hrs vs 48 hrs, *P* < 0.05), BAK (*P* < 0.001 vs. the respective control; 24 hrs vs. 48 hrs, *P* < 0.05), and BAX (*P* < 0.001 vs. the respective control; 24 hrs vs. 48 hrs, *P* < 0.05) in MCF-7 cells treated with 90 *μ*g/mL *C. ficifolia* extract for 24 and 48 hs were up-regulated as compared to control (Figure 6).

### 3.5. Effect of C. ficifolia Extract on DNA Damage

**Figure 7:**
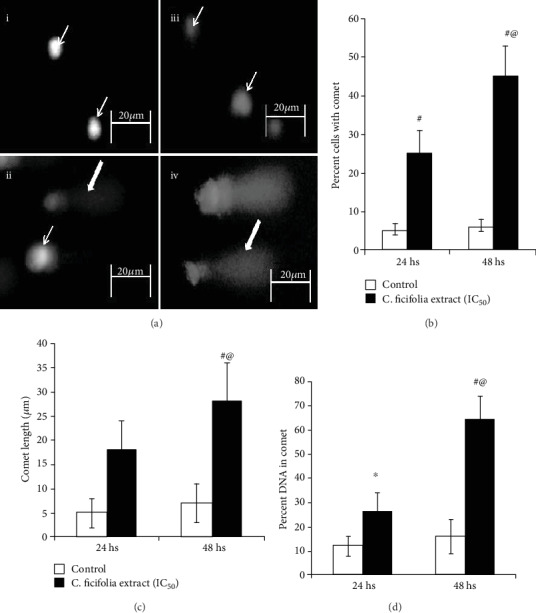
(a) DNA damage through comet assay. MCF-7 cells treated with *C*. *ficifolia* chloroform extract after 24 hs (i, ii) and 48 hs (iii, iv). (b) Percent cells with DNA damage (comet). Comet length (c) and percent DNA (d) in comet, respectively. Thin arrow shows cells without DNA damage. Thick arrow shows the cells with DNA damage (comet pattern). ^∗^*P* ≤ 0.05, ^#^*P* ≤ 0.001, respective control vs. treated samples. ^@^*P* ≤ 0.001, 24 hs treatment of samples vs. 48 hs treatment. The experiment was repeated thrice with triplicate.

Figure 7(a) shows the comet pattern and the percent of cells with comet, comet tail length, and percent of DNA in the head as shown in Figures 7(b)–7(d). Control cells at 24 hs and 48 hs showed a nil comet pattern (Figures 7(a), i and iii); on the other hand, cells treated with *C*. *ficifolia* showed statistically (*P* < 0.001) significant number of cells with DNA damage (Figures 7(a), ii and iv and Figure 7(b)). Tail length of treated cells (Figure 7(c)) is increased (*P* < 0.01) than that of untreated cells. Further, the increase in the duration of treatment augments the DNA damage (*P* < 0.001). Further, the results shows that control cells (Figure 7(d)) have higher percent of DNA in the head (>92%) and the tail region contains only ~5-7% DNA. For MCF-7 cells exposed with *C*. *ficifolia* alone (Figure 7(d)), the percent of DNA in the head region was decreased (*P* < 0.001) (~65% at 24 hs and~15% at 48 hs) and in the tail region was increased (*P* < 0.001) (~35% at 24 hs and ~85 at 48 hs) when compared to untreated MCF-7 cells.

## 4. Discussion

In many countries, pumpkin is used for its medicinal value as an antidiabetic as well as for the management of parasites and worms. In the past few decades, researchers have concentrated on the antidiabetic [25, 26], antihypertension [26], antibacterial, antifungal [27, 28] antihypercholesterolemic, intestinal antiparasitic [29], immunomodulatory, anti-inflammatory [30], and analgesic effects of pumpkin [31]. Polysaccharides present in the pumpkin are known to increase the cell immune function and are responsible for the immunomodulatory activity of pumpkin [32]. Furthermore, the augmentation of splenic lymphocyte proliferation, natural killer cell activity, and an elevation in the number of CD8+, CD4+ T cells, and the CD4+/CD8+ ratio have been observed in pumpkin extract-exposed animals [33]. Many investigators have explored the anticancer effect of pumpkin extracts [34, 35]. The cytotoxicity technique commonly has a wide range of understanding and is capable of sensing various novel anticancer compounds. Further, these compounds are capable of inhibiting the biochemical function in different human as well as animal cancer cell origin. Treatment with the *C*. *ficifolia* extract to the culture medium prevented the proliferation of MCF-7 cells in a dose- and time-dependent manner, revealing its cytotoxic potential. These findings agree that different *in vitro* investigations reported that pharmacologically effective compounds from natural sources proficiently decrease the viability of mammalian cancer cells through a wide mode of action [36, 37]. Normal hepatocytes were exposed to the *C*. *ficifolia* extract; it showed a higher IC_50_ value than that of MCF-7 cells. This study indicates the different compassion of the *C*. *ficifolia* extract on normal cells.

We observed a little but obvious elevation in DNA strand breaks by adopting the most sensitive technique such as TUNEL assay, in the extract of *C*. *ficifolia*-treated MCF-7 cells as compared to untreated cells. The *C*. *ficifolia* extract showed the morphological characteristics of apoptosis in MCF-7 cells. As observed by other investigators, TUNEL assay shows the type of DNA damage, which is essential for apoptosis in MCF-7 cells. These findings also denote that the TUNEL technique may have shown positive results for infrequent DNA damage. Similar observations were (large fragment formation) reported in other compound-induced cancer cytotoxicity-mediated cell death [38].

Normal healthy live cells become visible with uniformly green color stained with AO/EB, whereas early apoptotic cells appeared as green with a bright dot in the nuclei, which is due to the result of chromatin condensation. Late apoptotic cells also integrated the EB stain and henceforth were observed as orange, but, in distinction to necrotic cells, the late apoptotic cells were observed as condensed [39]. The treatment with the *C*. *ficifolia* extract induced a higher level of apoptotic death. Moreover, findings of the AO/EB staining confirmed that the extract of *C*. *ficifolia* brings about cytological changes such as cytoplasmic vacuolation, cytoplasmic blebbing, fragmentation of chromatin, bi-/multinucleation, late apoptosis indication of dot-like chromatin, and apoptotic body configuration. The current findings proved that the mode of cell death induced through a plant extract was apoptosis not through necrosis mechanisms [40].

Inhibiting cancer cell proliferation through an apoptotic mechanism becomes an obvious mode of action of the numerous anticancer compounds [41, 42]. Apoptotic event could be initiated through the extrinsic or intrinsic pathways [43]. The aspartate-specific group of cysteine protease called caspases plays a central mechanism in amending the apoptosis initiated by various types of stimuli, including oxidative stress [44]. Practically, caspase-3 is the central executor in the apoptotic event and caspase-9 is an originator of caspase-3 via the mitochondrial cytochrome c-mediated pathway [45]. There is enough evidence for the role of p53 in the regulation of the membrane expression of certain death receptors in several cell types [46]. A tumor suppressor protein such as p53 can also serve as a transcription factor. This protein regulates cell growth as well as cell death (apoptosis) in response to different modes of cellular anxiety or cell injury [47]. Functional loss of p53 weakens the response of cells to apoptotic motivation; this property is commonly observed in most of the human cancer cells. Thus, p53 appears to convey a signal to the apoptotic machines downstream of mitochondria [48]. In this study, we demonstrated that caspase-3, caspase-8, and caspase-9 and Tp53 expressions were significantly increased in cells treated with the *C*. *ficifolia* extract.

Fas ligand (FasL) induces apoptosis in a mode comparable to that of the TNF receptor. Interaction of the ligand develops receptor clustering, death-inducing signal complex development, and the commencement of the caspase cascade machinery. On the other hand, signaling through the Fas receptor is a little uncomplicated than through the TNF receptor. FADD, an adapter protein, could be directly employed to the death province of the Fas receptor, without involvement of the preemployment of the TRADD. Furthermore, the Fas receptor is commonly considered to just trigger apoptosis and activate the signaling [49]. In our results, we observed that the *C. ficifolia* treatment upregulated the expressions of Fas and FADD proteins leading to apoptosis of MCF-7 cells, which is mediated by caspase-8 interaction with FAS and FADD proteins.

The intrinsic (or mitochondrial) PCD cascade is initiated as a reaction to cellular stress. Proapoptotic BH3-only proteins are activated in the PCD intrinsic pathway. BAX and BAK are directly activated through BH3-only proteins by making its direct interactions with the above said protein; further, it can also activate through indirect mode by making an interaction with prosurvival Bcl-2-like proteins. Upon activation of BAX and BAK, these proteins forms oligomerization and helps to form pores in the mitochondrial outer membrane that release cytochrome c. Increased cytosolic cytochrome c concentration activates the caspases and causes succeeding cell death [50]. Diaz-Flores et al. [51] reported that *C*. *ficifolia* has an antioxidant effect on the glutathione redox cycle in mice with STZ-induced diabetes. In this study, treatment with the *C*. *ficifolia* fruit extract upregulated the expressions of proapoptotic BAX and BAK proteins, resulting in apoptosis of MCF-7 cells. It is crystal clear that DNA damage is one of the hallmarks of apoptosis [52]. Even though different techniques were available to assay DNA damage, comet assay is the one of most sensitive technique that detects the DNA damage at individual cells [53]. To validate the increased apoptotic gene expression in *C*. *ficifolia*-treated cells which leads to cellular DNA damage, hence, we have performed comet assay. This results shows that *C*. *ficifolia* induces the DNA damage. Numerous studies shows that well known anticancer drugs like cisplatin exerts its anticancer effect through DNA damage [54–56]. Thus, our study revealed that the *C*. *ficifolia* extract has chemotherapeutic potential against MCF-7 cells.

## 5. Conclusions

In this study, we have shown that *C*. *ficifolia* exercises its anticancer effect by inhibiting MCF-7 cell propagation. Further, it initiates the apoptotic process in part by altering the caspase-3, caspase-8, and caspase-9 and p53, FAS, FADD, BAX, and BAK gene expression levels. Henceforth, the current investigation through *C*. *ficifolia* significantly blocks the MCF-7 human breast cancer cell proliferation in laboratory condition and documents the probable mode of mechanism for its anticancer property. So, *C*. *ficifolia* could potentially stand for another source of medicine for breast cancer treatment. The current investigation supports the improvement of dietary plant substance-mediated drugs for the management of breast cancer. However, additional data are needed to sequence and identify specific molecules present in the *C*. *ficifolia* extract, which is responsible for its anticancer effect.

## Data Availability

The data used to support this study are included within the article.
